# Texas State Medical Association—Dr. F. E. Daniel’s Appointment as Secretary

**Published:** 1886-09

**Authors:** T. H. Nott

**Affiliations:** President Texas State Medical Association; Goliad, Texas


STATE MEDICAL ASSOCIATION.

*Dr. F. E. Daniel, Austin, Texas:*

DEAR DOCTOR—Enclosed please find your appointment to the Secretaryship of our State Association to fill the vacancy caused by the death of our lamented Burt. I hope it will suit you to accept the appointment, as it is the result of due deliberation and consultation, and also of many voluntary letters written to me, asking your appointment.

Respectfully,

T. H. NOTT, M. D.,

President Texas State Medical Association.

GOLIAD, Texas, July 30, 1886.

